# Let-7 inhibits self-renewal of hepatocellular cancer stem-like cells through regulating the epithelial-mesenchymal transition and the Wnt signaling pathway

**DOI:** 10.1186/s12885-016-2904-y

**Published:** 2016-11-08

**Authors:** Bin Jin, Wei Wang, Xiang-xin Meng, Gang Du, Jia Li, Shi-zhe Zhang, Bing-hai Zhou, Zhi-hao Fu

**Affiliations:** 1 Department of General Surgery, Qilu Hospital of Shandong University, 107 Wenhua West Road, Lixia District, Jinan, Shandong Province 250012 China; 2 Department of Biochemistry and Molecular Biology, School of Medicine, Shandong University, Jinan, Shandong Province 250012 China; 3 Department of General Surgery, The People’s Hospital of LingCheng, Dezhou, 253500 China

**Keywords:** Let-7 miRNAs, Hepatocellular carcinoma, Cancer stem-like cells, EMT, Wnt signaling

## Abstract

**Background:**

Tumor suppressive let-7 miRNAs are universally down-regulated in human hepatocellular carcinoma (HCC) versus normal tissues; however, the roles and related molecular mechanisms of let-7 in HCC stem cells are poorly understood.

**Methods:**

We examined the inhibitory effect of let-7 miRNAs on the proliferation of MHCC97-H and HCCLM3 hepatic cancer cells by using MTT (3-(4,5-dimethylthiazol-2-yl)-2,5-diphenyltetrazolium bromide) assay, which was further confirmed by apoptosis and cell cycle studies. The sphere-forming assay was used to study the effects of let-7a on stem like cells. Through western blot, immunofluorescence and the luciferase-reporter assay, we explored the activity of epithelial-mesenchymal transition (EMT) signaling factors in HCC cells. qRT-PCR was applied to detect miRNA expression levels in clinical tissues.

**Results:**

Let-7a effectively repressed cell proliferation and viability, and in stem-like cells, also let-7a decreased the efficiency of sphere formation.in stem-like cells. The suppression of EMT signaling factors in HCC cells contributed to let-7’s induced tumor viability repression and Wnt activation repression. Besides, Wnt1 is critical and essential for let-7a functions, and the rescue with recombinant Wnt1 agent abolished the suppressive roles of let-7a on hepatospheres. In clinical HCC and normal tissues, let-7a expression was inversely correlated with Wnt1 expression.

**Conclusions:**

Let-7 miRNAs, especially let-7a, will be a promising therapeutic strategy in the treatment of HCC through eliminating HCC stem cells, which could be achieved by their inhibitory effect on the Wnt signaling pathway.

## Background

Hepatocellular carcinoma (HCC) is one of the most aggressive malignancies worldwide, being recognized as the third leading cause of cancer-related deaths [1, 2]. HCC is the fifth most common malignancy in men and the seventh among women [3]. The incidence of HCC depends on geography, and most of the burden is in developing countries, occurred with hepatitis. The situation is more severe in China, with poorer 5-year survival [4]. The tumorigenesis of HCC is a multistage process including noncoding and protein-coding genes. MiRNAs are found to be deregulated in most malignancies, affecting carcinogenesis, progression, metastasis and tumor recurrence. In HCC, it has been reported that aberrant expression of let-7 miRNAs contributed to the development and progression of HCC [5, 6].

Research has indicated that some miRNAs may function as oncogenes when up-regulated in HCC; to the contrary, the down-regulated miRNAs suggested them as tumor suppressors [7, 8]. Functioning as tumor suppressors, let-7 miRNAs were found to repress Ras, Bcl-xl, MAPK, c-Myc, cyclin D1 and other oncogenes in HCC [8, 9]. Wnt1 stimulates the Wnt/β-catenin/TCF pathway, leading to different cell fates, and then regulating the transcription of many downstream genes which contain the TCF/LEF1 motif, affecting biological functions and the maintenance of self-renewal of cancer stem cells (CSCs) [10–12]. However, there is no research focused on the relationship between let-7 and the Wnt signaling pathway.

Tumors consist of cells with heterogeneity, with different characteristics, and the CSCs are demonstrated to be steady and stable through clinical chemo-radiotherapy, due to the slow cell cycles and low proliferative ability, contributing to tumor relapse and occurrence of resistance [8, 13]. Therefore, treatments targeting these silent CSCs will show great potential in eliminating the tumor group entirely, helping to overcome resistance to therapy and recurrence of the tumor. However, the underlying mechanism by which let-7 works to inhibit CSCs in HCC remains largely unknown.

## Methods

### Cell culture, transfection and infection

MHCC97-H and HCCLM3 human HCC cells were purchased from ATCC and maintained at the central laboratory of the Qilu Hospital of Shandong University. The cells are cultured in DMEM medium (Invitrogen, USA), containing 10 % fetal bovine serum (FBS), 1 % penicillin and 1 % streptomycin (Invitrogen, USA). The spheres (hepatospheres) were cultured in DMEM/Ham’s F-12 medium compounded with 10 ng/ml epidermal growth factor, 10 ng/ml human basic fibroblast growth factor, 4 μg/ml insulin, 1 % penicillin and 1 % streptomycin (Invitrogen, USA). Oligonucleotides encoding mature let-7a/b/c/d/e/f/g/i miRNAs and miRNA-LSC1 were synthesized by Invitrogen and cloned into the lentiviral vector lentilox3.7 (pLL3.7). SiRNAs targeting Wnt1were synthesized and purchased from GenePharma Inc. (Shanghai, China). Transfections were performed using Lipofectamine 2000 (Invitrogen, USA), and the siRNAs were added the second days after cells were plated. Transiently transfected HCC cells were harvested 48 h post-transfection.

### Reverse transcription PCR and real-time PCR

Total RNA was isolated from fresh clinical specimen using TRIzol® Reagent (Invitrogen, USA) following mechanical tissue homogenization, or from or cultured cells using Trizol reagent after treatment with 5 μg/ml puromycin for 48 h. Approximately 1 μg of RNA was reverse-transcribed to single strand cDNA using PrimeScript RT Master Mix (Takara Biotechnology, China). The real-time PCR was conducted in a total volume of 25 μl as previously reported [7, 14]. The cycle threshold (Ct) was automatically calculated by iQ™-5 Optical Module software (Bio-rad, USA). The relative expression of let-7 family members was normalized to U6 expression, and then calculated using the formula 2^−ΔΔCt^ method, versus the scramble control.

### Sphere formation assays

The spheres formation assay is often used to enrich stem-like cells, being are used to isolate and expand population enriched in CSCs [15–18]. Cells of different groups were seeded in ultra-low adherent-conditioned plates (Corning, USA) to test their ability of forming primary spheres, in the presence of puromycin. In total, 12,000 cells were seeded per 6 cm plate. On day 9, cell sphere number of spheres was counted using an inverted microscope, a sphere was defined as contaning more than 10 cells. The sphere-forming efficiency was calculated as the percentage of counted spheres versus seeded cells [19]. Spheres of different groups were disaggregated, and 12,000 cells were then re-suspended per 6 cm plate to test their self-renewal ability.

### Immunofluorescence and IHC

Cells were seeded on glass chamber slides for 24 h, and then fixed by 10 % formalin for 15 min. Antigens were blocked with 2 % normal goat serum (ab7481, Abcam, USA), cells werethen incubated with the β-catenin antibody for 1 h in PBS, and then incubated with Alexa Fluor® 488 (Life technologies, USA) for 30 min, washed in PBS, incubated for 10 min with 2 μg/ml of Hoechst 33342 (Life technologies, USA), and washed with PBS again. For IHC, the paraffin tissues were rehydrated in an alcohol gradient and then were rinsed in deionized water. Endogenous peroxidase activity was blocked using a 0.03 % hydrogen peroxide solution. 1:200 IHC-TekTM Antibody Diluent (IW-1000, Ellicott City, MD, USA) was used to reduce background staining. The section was then incubated with 1 μg/ml ab16051 for 1 h at room temperature and detected using an HRP, DAB was used as the chromogen.

### Flow cytometry analysis

The propidium iodide (PI) and Annexin V-FITC (Green) kits were obtained from BD Biosciences (San Jose, CA). Cells of different groups were collected and trypsinized into a single cell, and then washed twice with PBS. All cells were resuspended in 500 μl binding buffer respectively, and 5 μl of Annexin V-FITC and 10 μl of PI were added. The mixture was gently vortexed and then incubated for 15 min at room temperature in the dark. The cells were analyzed by FACSAria cytometry within 1 h of incubation using BD FACSuite Software. For cell cycle analysis, 5 × 10^5^ cells were collected and washed twice with PBS. Cells were fixed with ice-cold 70 % ethanol at 4 °C for 24 h. Before detection, the fixed cells were stained with PI for 30 min at 37 °C, followed by FACSAria cytometry. All tests were performed in triplicate. Cell cycle analysis of DNA content was performed using MultiCycle software. Instrument Setup instructions were followed as presenteding online documentation: https://www.bdbiosciences.com/documents/BD_FACSVerse_Apoptosis_Detection_AppNote.pdf.

### Luciferase assay

The TCF/lymphoid enhancer factor luciferase reporter (TOP) and its negative control (FOP) plasmids were obtained from Upstate Biotechnology (NY, USA). Cells were plated at 30–40 % confluence in 24-well plates 16 h prior to transfection by using FuGENE 6 (Roche, USA) [20].

### Let-7 and Wnt1 expression in clinical tissues

To explore the role of let-7a in HCC, we first collected specimens from 58 patients, who underwent surgery at the Department of General Surgery, the Second Hospital of Jilin University from June 2008 to November 2013. Of the samples collected, 20 slides were prepared for IHC using a Wnt 1 (1:200; sc-6280, Santa Cruz, USA). All of the patients were diagnosed and confirmed by pathological examination. No preoperative chemotherapy or radiotherapy was performed in any of these patients. Specimens were stored in liquid nitrogen. This study was conducted under the supervision of the Ethical Board of Jilin University.

### Statistical analysis

All data were obtained from at least three independent experiments, are expressed as mean ± SD and were analyzed by Student’s *T*-test and *χ* ^2^ test using SPSS for Windows version 16.0 (IBM, USA) and Excel 2010 (Microsoft, USA).

## Results

### The function of let-7 miRNAs in HCC cells

**Fig. 1:**
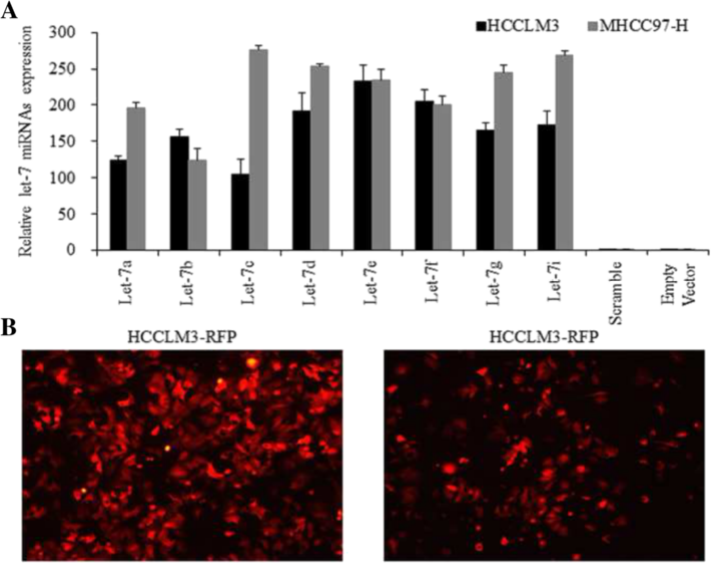
The construction of let-7 miRNAs overexpressing HCC cells. **a**. The expression levels of let-7 miRNAs in HCC cells were detected after lentivirals infection, and results showed that we successfully constructed let-7 overexpressing HCC cells. The lentivirals vectors infected cells were *red* when observed under Inversed Fluorescent Microscope

**Fig. 2:**
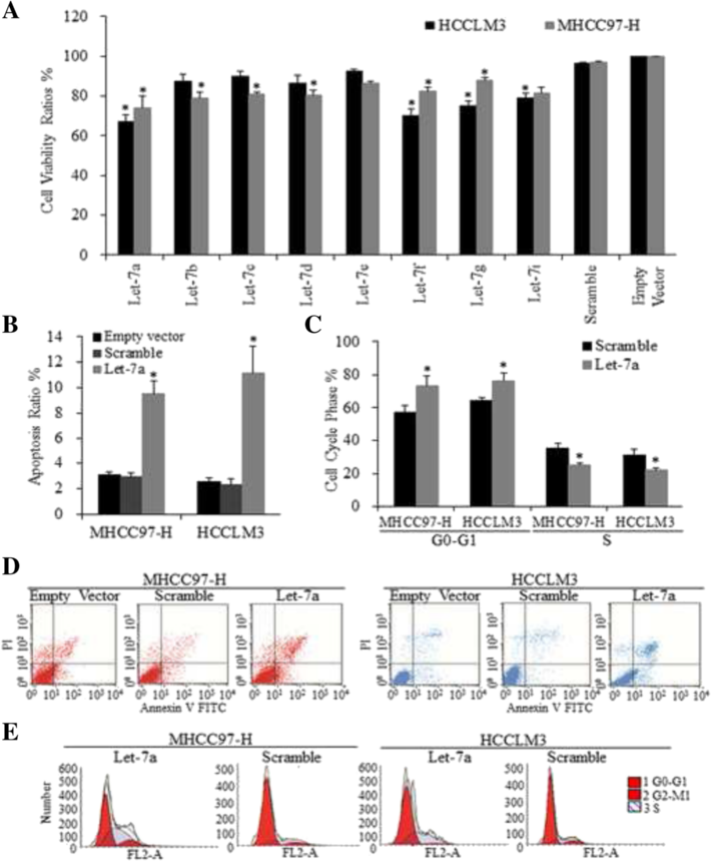
The inhibitory effects let-7 on HCC cells. **a**. After let-7 miRNAs were successfully overexpressing in HCC cells, the effects of let-7 on cell proliferation were detected by MTT assay. Let-7a was demonstrated to exert the strongest repression on cell proliferation, defined by student *t* test and Two-way ANOVA, * *p* < 0.01. Let-7a induced more cell apoptosis in MHCC97-H and HCCLM3 cells (**b**); let-7a also increased the portion of cells staying in G0-G1 stage and decreased the cells in S phase (**c**), compared to Scramble group. * *p* < 0.01. The representative images of the FACS derived apoptosis ratios (**d**) and FACS derived cell cycle analysis (**e**)

RFP based let-7a/b/c/d/e/f/g/i lentiviral vectors were successfully infected into HCC cells, asshown in Fig. 1. Inhibition of cell proliferation was detected using MTT assay at 48 h. The proliferation of let-7a-overexpressing HCC cells was suppressed most effectively compared to scramble and empty vector groups, as determined by the Student’s *T*-test (*p* < 0.01) and ANOVA (*p* < 0.01) analysis with Bonferroni correction (Fig. 2a).

### Overexpressing let-7a induced apoptosis and cell cycle arrest of HCC cells

To examine whether the enforced let-7a could induce cell apoptosis and cell cycle arrest, both MHCC97-H and HCCLM3 cell lines were subjected to Flow cytometry analysis. We found that both of these HCC cell lines exhibited higher apoptosis ratios (*T*-test, *p* < 0.01, Fig. 2b). Let-7a also induced cell cycles arrest at G1 (*T*-test, *p* < 0.01, Fig. 2c). Representative images of apoptosis and cell cycle are shown in Fig. 2d-e.

### Let-7a suppressed the sphere formation efficiency of HCC cells

**Fig. 3:**
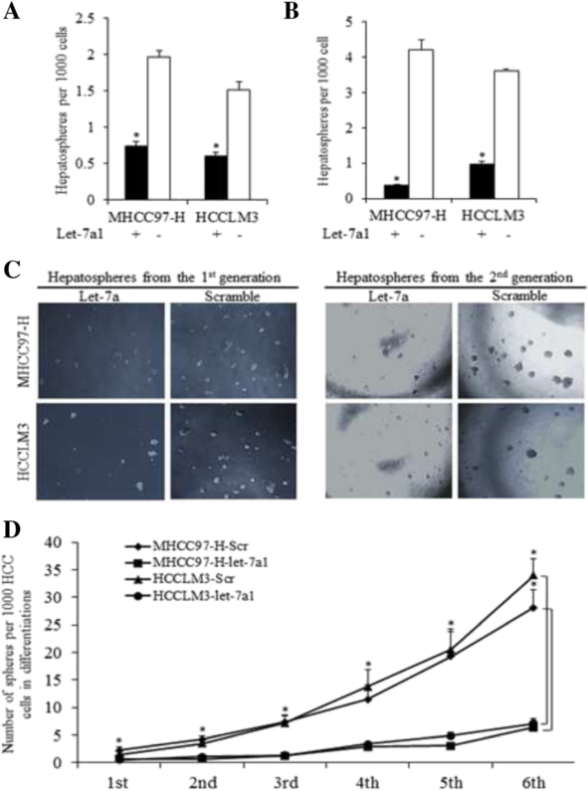
Let-7a1 inhibits the capacity of self-renewal of HCC stem cells. The HCC cells were seeded in ultra-low attachment plates to form the 1st generation of spheres. Then the cell form spheres were reseeded to acquire the 2nd generation of spheres. The sphere forming efficiency of the first generation (**a**) and the second generation (**b**) of HCC stem-like cells infected with let-7a1 and Scramble vector, * *p* < 0.01, with representative images shown in (**c**). **d** Let-7a1 inhibits the self-renewal of HCC stem cells group in continuously cultured spheres, showing much stronger inhibition than that of control group in total six generations

Sphere formation efficiency of MHCC97-H-let-7a and HCCLM3-let-7a cells was significantly lower than that of Scramble groups (*T*-test, *p* < 0.01, Fig. 3a). Disaggregation of primary hepatospheres and secondary plating of suspending cells led to the formation of hepatospheres again, and the sphere forming efficiency of MHCC97-H-let-7a and HCCLM3-let-7a secondary spheres was lower than that of Scramble groups (*T*-test, *p* < 0.01,Fig. 3b), as were shown in Fig. 3c. To further confirm the effects of let-7a1 on HCC stem cells, continuous sphere culture assay was applied, and up to six generations were cultured and detected. Let-7a1 showed significant impacts on HCC stem cells, and the inhibitive influence could be accumulated (Fig. 3d).

### Overexpressing let-7a suppressed the EMT factors of HCC cells and Wnt signaling pathway of HCC stem-like cells

**Fig. 4:**
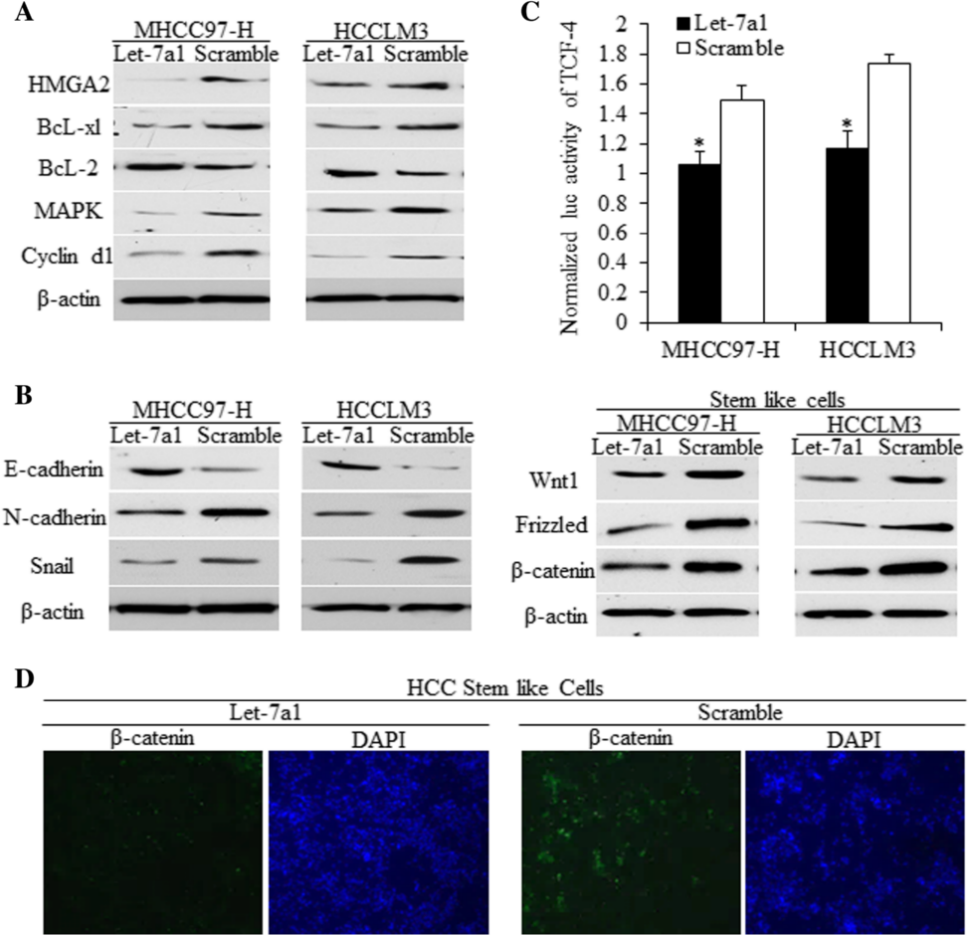
The suppressions of EMT factors of HCC cells and Wnt activation of HCC stem cells were related to let-7a functions. **a**. Gene expression levels of let-7 targeted genes, which were implicated in cell apoptosis and cell cycle regulations. Let-7a decreased HMGA2, Bcl-xl, MAPK, cyclin d1, and increased Bcl-2 in both cell lines. **b**. Genes related to EMT were detected, and results showed that let-7 inhibited the EMT of HCC cells through regulating E-cadherin, N-cadherin and Snail, of which, E-cadherin was up-regulated, whereas, the mesenchymal biomarker N-cadherin and Snail were decreased. **c**. Let-7a1 inhibited the TCF-4 promoter activity of HCC cells, and inhibited Wnt1/Frizzled/β-catenin signaling of HCC stem cells, which was demonstrated to promote the self-renewal ability of cancer stem cells. **d**. The results of immunofluorescence showed that β-catenin was decreased due to let-7 overexpression in HCC stem cells

We first detected markers related to apoptosis and cell cycle (Fig. 4a). To explore the possible mechanisms by which let-7a represses sphere number, we hypothesized that increased let-7a in HCC cells and HCC stem cells inhibited malignant cellular behaviors through down-regulating N-cadherin and Snail, that has been typical pathological markers for epithelial trait was up-regulated; meanwhile, was involved in the process of epithelial-mesenchymal transition (EMT) (Fig. 4b, Left). In HCC stem-like cells, key molecules of the Wnt signaling pathway universally decreased due to overexpressing let-7a, indicating that let-7a inhibited the Wnt1/Frizzled/β-catenin pathway in a population enriched with HCC stem cells (Fig. 4b, right). Further, using the luc-reporter assay and immunofluorescence staining, we found that let-7a inhibited Wnt signaling, which was achieved by decreasing TCF-4 promoter activity (*T*-test, *p* < 0.01, Fig. 4c) and β-catenin expression (Fig. 4d).

### Decreased Wnt1 is required for let-7a-induced renewal inhibition of hepatospheres

**Fig. 5:**
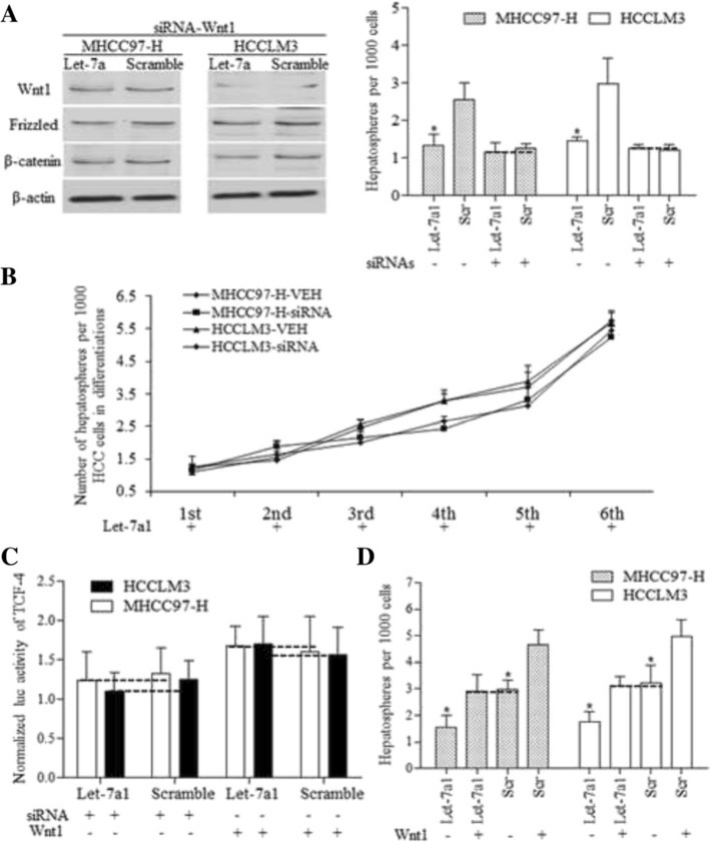
Let-7a inhibits the HCC spheres through Wnt1. **a**. The knockdown of Wnt1 (left) decreased the sphere forming efficiency of HCC stem cells, and abolished the functions of let-7a on self-renewal ability (right), which functioned through regulations of Wnt1/Frizzled/β-catenin pathway (left). **b**. In continuous culture of HCC spheres, the Wnt1 siRNA didn’t affect the self-renewal ability of let-7a1 overexpressing HCC stem-like cells, compared to Control group (VEH). **c**. The knockdown of Wnt1 decreased TCF-4 activity, abolished let-7a effects; similarly, the addition of Wnt1 reversed let-7a effects, increasing TCF-4 activity, and no significant differences between let-7a and Scramble group were detected. **d**. The addition of Wnt1 reversed the suppressive effects of let-7a on MFE

We used the RNA interference assay to suppress Wnt1 activity in HCC cells by three independent siRNA. As with let-7a1 overexpression, knockdown of Wnt1 significantly inhibited the self-renewal ability of stem-like cells, and inhibited Wnt signaling pathway factors (Fig. 5a). In the presence of Wnt1 siRNA, let-7a1 did not show significant inhibition on sphere formation ability in continuous stem cell culture compared to the scramble control (Fig. 5b). The knockdown of Wnt1 decreased TCF-4 activity significantly, abolishing let-7a functions (Fig. 5c). To identify whether Wnt1 is critical for let-7a-repressed self-renewal ability of HCC stem cells, MHCC97-H and HCCLM3 cells were cultured in ultralow attachment plates with recombinant Wnt1 protein (50 ng/ml) for 8 days. The addition of recombinant Wnt1 protein increased the sphere formation efficiency of both let-7a1 and scramble controls, reversing the suppressive roles of let-7a (Fig. 5d), and also increased TCF-4 activity (Fig. 5c).

### Let-7a sensitized HCC stem-like cells to cis-platinum-induced self-renewal inhibition

**Fig. 6:**
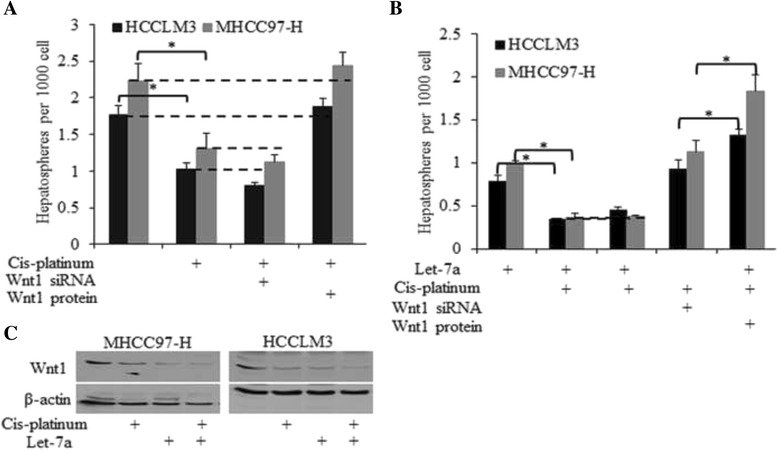
Let-7a sensitized HCC stem-like cells to cis-platinum induced self-renewal inhibition. **a**. Cis-platinum reduced sphere number through inhibition of Wnt1 expression. **b**. The combined use of cis-platinum and let-7a significantly decreased the sphere number, inhibiting the self-renewal ability of HCC stem-like cells through concurrent effects on Wnt signaling. **c**. Both let-7a and cis-platinum inhibited Wnt1 explression level, and exerted sygnergic inhibition on Wnt1 activity

Cis-platinum is one of the most commonly used anticancer drugs in clinical treatment; however its role in the regulation of CSCs is not well understood. We found that cis-platinum reduced spheres number (Fig. 6a-b) through the inhibition of Wnt1 expression (Fig. 6c); the effects were reversed by recombinant Wnt1 protein (Fig. 6a). The combined use of cis-platinum and let-7a significantly decreased the sphere number, inhibiting the self-renewal ability of HCC stem-like cells through concurrent effects on Wnt signaling.

### The loss of let-7a was related to the occurrence and progress of HCC

**Fig. 7:**
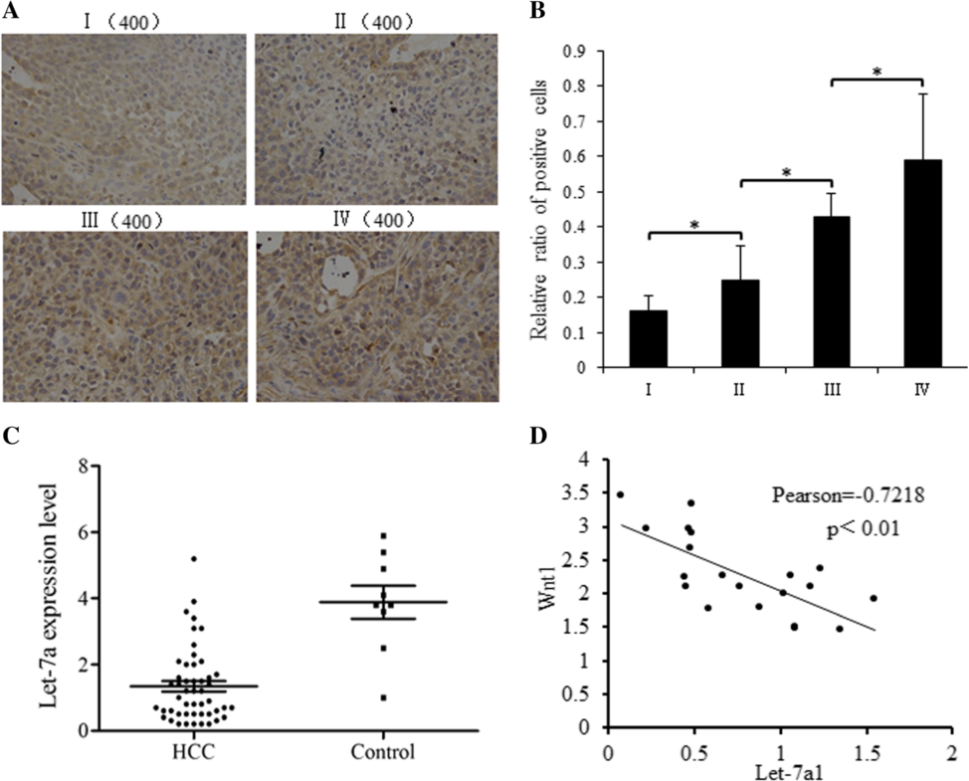
Let-7a was inversely related with Wnt1 and indicated better clinical prognosis. **a**. Representative images of immunohistochemical staining for β-catenin in tissues of different clinical stages. **b**. **d**. Quantification of relative immunostaining intensity of β-catenin, StagingIhad the least expression of β-catenin. **c**. Quantification of let-7a in HCC tissues and adjacent normal tissues, the relative quantity was calculated by 2^-ΔΔCt^, with U6 acting as the internal reference. The statistical analysis was performed using *T* test. **d**. Wnt1 mRNA expression in tissues from 20 patients were tested, and there is inverse correlationship between let-7a miRNA and Wnt1 mRNA was found, Pearson = −0.722, *p* < 0.01

IHC studies show that the level of β-catenin was much higher in tumors with later clinical stages (Fig. 7a-b). In clinical HCC samples, let-7a expression was much lower in tumor tissues than adjacent normal tissue (Fig. 7c), indicating an inverse relationship between let-7a expression and HCC occurrence. Likewise, let-7a expression level was inversely correlated with Wnt1 mRNA in HCC tissues (Fig. 7d).

## Discussion

Let-7 is a family consisting of 13 members located on nine different chromosomes whose expression is usually lost, reduced, or deregulated in most human malignancies [21]. Growing evidence suggests that the restoration of let-7 expression effectively repressed cell proliferation, invasion, metastasis, and resistance to therapy. The findings of let-7 repression on CSC self-renewal indicated that let-7 restoration may be a useful therapeutic option in HCC and stem-like cells, which was more crucial for curing the cancer [8, 22–24]. Recent studies found that cholesterol-conjugated let-7a inhibited cell proliferation, growth, and metastasis, and mainly functioned in the cytoplasm through directly reaching HCC orthotropic tumors [25]. What’s more, the therapeutic trial of let-7 mimics showed suppressed effects on tumor growth in pre-clinical studies [4]. Especially, nanoparticle-based let-7 replacement therapy had been successfully applied in vivo, together with other delivery methods, including lentivirus-mediated pre–let-7 s, adenovirus-mediated hairpin sequences of mature let-7, cationic liposome–mediated pre–let-7, and electroporation of synthetic let-7 [8, 26].

In this study, we show that overexpressing let-7a exerted inhibitory effects on HCC, consistent with previously published results for other malignancies [27, 28]. EMT inducers, including Snail, Slug, Twist1, ZEB1 and ZEB2, suppress the expression of adherence proteins to induce cellular malignancies. EMT is a major mechanism for cancer generation, metastasis and progression [8], which ultimately promote the growth of tumor bulk and cell proliferation, and during the EMT process, CSCs are generated [29]. We found that increased let-7a could inhibit sphere formation efficiency through alleviating EMT via down-regulating N-cadherin and Snail in HCC cells. In HCC stem-like cells, overexpressing let-7a inhibited the Wnt1/Frizzled/β-catenin signaling pathway, which was involved in maintaining the self-renewal ability of stem cells. We further identified that repressed Wnt1/Frizzled/β-catenin signaling in a CSC-enriched population was attributed to enforced let-7 and let-7 enhanced cis-platinum functions, helping to inhibit the self-renewal of stem-like cells. Our results suggest that overexpression of let-7a could be used as a therapeutic agent and prognostic indicator in the management of HCC against Wnt activation, and help to understand the mechanisms through which let-7 regulated HCC stem cells.

Let-7 functions are detailed explored in many kinds of tumors, and let-7 acted through post-transcriptional regulations of the targeted genes [30]. However, the roles of let-7 in HCC stem-like cells are less involved. For the first time, we identified the let-7 controlled Wnt signaling activity, which was accused for maintaining of cell pluripotency. Wnt/β-catenin transactivation of let-7 in breast cancer further suggested the regulatory roles of let-7 in stem cells’ regulations [31]. Overall, our results suggest that overexpression of let-7a could be used as a therapeutic agent and prognostic indicator in the management of HCC via repression of Wnt signaling activation in stem cells, and to help understand the mechanisms through which let-7 regulates HCC stem cells.
